# Color Stability and Surface Properties of PMMA/ZrO_2_ Nanocomposite Denture Base Material after Using Denture Cleanser

**DOI:** 10.1155/2021/6668577

**Published:** 2021-04-07

**Authors:** Mohammed M. Gad, Reem Abualsaud, Shaimaa M. Fouda, Ahmed Rahoma, Ahmad M. Al-Thobity, Soban Q. Khan, Sultan Akhtar, Khalid S. Al-Abidi, Mohamed S. Ali, Fahad A. Al-Harbi

**Affiliations:** ^1^Department of Substitutive Dental Sciences, College of Dentistry, Imam Abdulrahman Bin Faisal University, P.O. Box 1982, Dammam 31441, Saudi Arabia; ^2^Department of Restorative Dental Sciences, College of Dentistry, Imam Abdulrahman Bin Faisal University, P.O. Box 1982, Dammam 31441, Saudi Arabia; ^3^Department of Clinical Affairs, College of Dentistry, Imam Abdulrahman Bin Faisal University, P.O. Box 1982, Dammam 31441, Saudi Arabia; ^4^Department of Biophysics, Institute for Research and Medical Consultations, Imam Abdulrahman Bin Faisal University, P.O. Box 1982, Dammam 31441, Saudi Arabia

## Abstract

**Objectives:**

This study aimed to evaluate denture cleanser effects on color stability, surface roughness, and hardness of PMMA denture base resin reinforced with nano-ZrO_2_.

**Materials and Methods:**

A total of 420 specimens were fabricated of unreinforced and nano-ZrO_2_ reinforced acrylic resin at 2.5% and 5%, resulting in 3 main groups. These groups were further subdivided (*n* = 10) according to immersion solution (distilled water, Corega, sodium hypochlorite, and Renew) and immersion duration. Surface roughness, hardness, and color were measured at baseline (2 days-*T*_0_) in distilled water and then after 180 and 365 days of immersion (*T*_1_ & *T*_2_) in water or denture cleansing solutions. Data was collected and analyzed using two-way ANOVA followed by Bonferroni post hoc test (*α* = 0.05).

**Results:**

Surface roughness increased significantly after denture cleanser immersion of unmodified and nano-ZrO_2_-modified PMMA materials while hardness decreased (*P* < 0.001). The denture cleansers significantly affected the color of both PMMA denture bases (*P* < 0.001). The immersion time in denture cleansers significantly affected all tested properties (*P* < 0.001). Within denture cleansers, NaOCl showed the highest adverse effects (*P* < 0.05) while Renew showed the least adverse effects.

**Conclusion:**

Denture cleansers can significantly result in color change and alter the surface roughness and hardness of denture base resin even with ZrO_2_ nanoparticles addition. Therefore, they should be carefully used.

## 1. Introduction

Although polymethyl methacrylate (PMMA) is the most common material used for denture base fabrication, it possesses low surface properties that enhance the attachment of *Candida albicans* (*C. albicans*), which is considered the prime pathogen of denture-induced stomatitis [1]. Therefore, denture cleansers (DCs) have been suggested as means for denture care and maintenance protocols [2]. An ideal denture cleanser must be biocompatible, microbicidal, harmless to the denture, effective in removing all deposits, and easy to use. When choosing the DC, one should consider its biocompatibility with the material being disinfected [3]. However, studies have shown that daily use of DCs can affect the physical and mechanical properties of denture base material [4–7]. DCs contain one or more active ingredients, such as alkaline peroxides, sodium hypochlorite (NaOCl), chlorhexidine, or enzymes [4, 5].

The denture properties that might be affected include surface roughness and hardness which are essential for the long-term success of dental prostheses [5, 6]. Previous studies reported the role of surface roughness, irregularities, porosities or indentations in plaque formation, and denture staining compared to smooth surfaces [7]. A satisfactory level of surface hardness is important to resist surface changes brought up by cleaning during denture lifetime [7, 8]. Change in color of denture base resins is one of the signs of aging [9]. Resin material with its ability to absorb liquids or dissolute over time may stain or change color after prolonged use [9]. Previous studies deduced that immersing in denture cleansers could alter the color and increase the surface roughness of heat-polymerized denture base resin [4, 10].

Recent trends are directed toward nanoparticle incorporation into other materials' structures to produce nanocomposites with reasonable properties. For example, zirconium dioxide nanoparticles (nano-ZrO_2_) are commonly used for acrylic resin reinforcement [11]. This popularity was due to their properties such as biocompatibility, high strength, and esthetic acceptability [11]. Earlier studies proved that 2.5–5% nano-ZrO_2_ addition improved the mechanical and physical properties of PMMA/ZrO_2_ nanocomposite, and the final properties were dependent on the concentration of the nanofiller [11–14]. However, the effect of denture cleansers on color stability, surface roughness, and hardness of PMMA/ZrO_2_ nanocomposite denture base material has not yet been investigated. Therefore, this *in vitro* study aimed to assess the effects of DCs on color stability, surface roughness, and hardness of PMMA/ZrO_2_ nanocomposite compared to the unmodified version of the material. The null hypothesis is that DCs will not affect surface roughness, hardness, or the color of PMMA/ZrO_2_ denture base material.

## 2. Materials and Methods

### 2.1. Specimens Preparation

**Figure 1:**
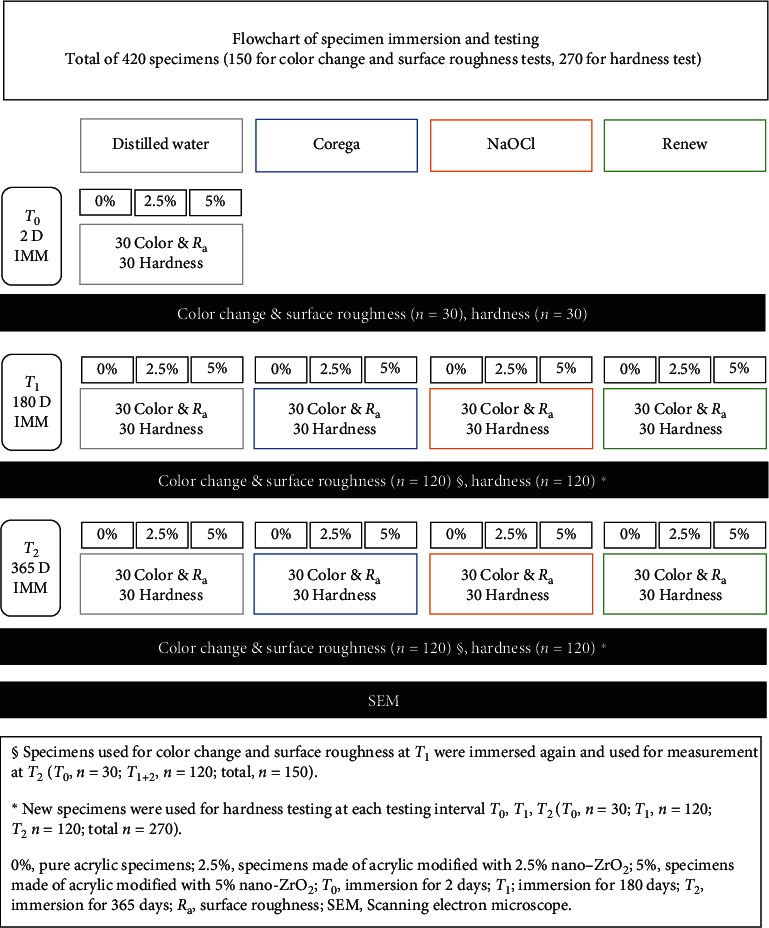
Flowchart of specimen immersion and testing.

Similar to the process described in previous studies [12–14], for PMMA/ZrO_2_ nanocomposite preparation, nano-ZrO_2_ powder (99.9%, <100 nm, 1314-23-4, Shanghai Richem International Co., Ltd.) was treated with a silane coupling agent (3-trimethoxysilyl propyl methacrylate, 97%) (*γ*-MPS) (Shanghai Richem International, Shanghai, China) and added to the powder of heat-polymerized acrylic resin (Major Base 20, Prodotti Dentari SPA, Moncalieri, Italy) in the concentration of 2.5 wt% and 5 wt%, dividing the specimens into 3 groups according to the filler percentage. Further subdivisions were done according to immersion solution and duration (*n* = 10) (Figure 1). A total of 420 acrylic disc specimens (15 × 2 mm, 270 for hardness test and 150 for surface roughness and color change tests) were fabricated from the heat-polymerized acrylic resin using the conventional technique of complete denture fabrication and following the manufacturer's instructions, and as described in previous studies [2, 13]. After polymerization, a single operator finished the divested specimens in wet conditions using 120 to 500-grit alumina abrasive discs and a polishing machine (Metaserve 250 grinder/polisher, Buehler) followed by polishing with a felt disc.

### 2.2. Denture Cleansers Preparation and Immersion Protocol

**Figure 2:**
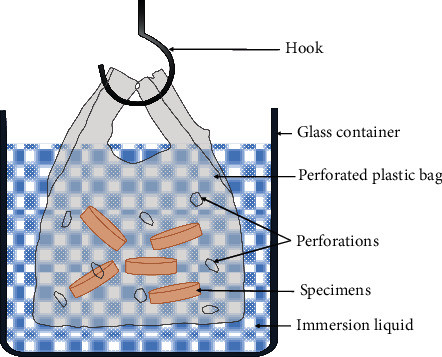
Illustrated diagram for specimen immersion in denture cleansers.

DCs used in the current study with their chemical composition, preparation, and immersion duration are summarized in Table 1.  Figure 2 shows a schematic representation of the immersion procedure.

### 2.3. Color Stability Test

For color analysis, color measurements were recorded with the help of a reflectance spectrophotometer (Color-Eye® 7000A, X-Rite) and CIE *L*^*∗*^*a*^*∗*^*b*^*∗*^ color scale (Commission Internationale de I'Eclairage) using standard illuminant (D65) in the wavelength range of 360–740 nm. The three color coordinates were recorded at each time interval, and Δ*E* was calculated (between baseline and different immersion durations) using the formula Δ*E*^*∗*^=[(Δ*L*^*∗*^)^2^+(Δ*a*^*∗*^)^2^+(Δ*b*^*∗*^)^2^]^1/2^ [2, 15]. The mean values were compared in terms of tested material, aging methods (immersion solution), and duration. Color changes (Δ*E*) were converted to National Bureau of Standards (NBS) units [10, 15] using the formula: NBS units = Δ*E* × 0.92 and correlated with the corresponding meaning [15, 16].

### 2.4. Surface Roughness

Surface roughness measurements were performed using a noncontact profilometer (Contour GT, Bruker Nano gmbH Schwarzschildstrasse 12, 12489 Berlin, Germany). The specimens were removed from immersion solutions at different time intervals (*T*_0_, *T*_1_, *T*_2_), rinsed under running water, dried using absorbent paper, and evaluated for surface roughness (*R*_a_, *μ*m) at three areas, 0.8 mm apart at the center of the specimen.

### 2.5. Hardness Test

Hardness (Vickers Hardness Number; VHN) test was performed by a single operator at each time interval using a hardness tester (Wilson Hardness, ITW Test & Measurement GmbH, Shanghai, China). Five indentations at 100 gf and 30 s dwell time were made per specimen, and the average of these readings represented the final hardness value for that specimen [17].

### 2.6. Scanning Electron Microscopy (SEM) Analysis

For scanning electron microscopy (SEM) analysis, selected specimens at each time interval were dried at 37°C for 24 h, gold sputter-coated (Quorum, Q150 R ES), and then examined by SEM (FEI, Inspect S50, Czech Republic, 20 kV) in order to study the effects of the solutions on the surface of the specimens.

### 2.7. Statistical Analysis

Statistical Package for Social Sciences (SPSS version 23) was used for data entry and analysis. The normality of data was checked through the Shapiro-Wilk test. In inferential statistics, one-way ANOVA was used to find the effect of concentration and time independently on each tested property. Pairwise analyses of different levels of concentration and time were done through the Bonferroni post hoc test for significant *P* value. Two-way ANOVA was employed to test the combined effect of time and concentration over the various properties of specimens tested. Statistical significance was set at *P* ≤ 0.05.

## 3. Results

Table 2 shows the variation in color, surface hardness, and roughness between different DCs at each nanofiller concentration and immersion duration. The means for all tested properties were found statistically significant at each level of concentration and time (*P* < 0.001). Furthermore, it was observed that the calculated Δ*E* and surface roughness values were the least for distilled water followed by Renew and then Corega. NaOCl showed the highest Δ*E* and surface roughness values among all solutions. The same pattern was observed at each nano-ZrO_2_ concentration and immersion time. For hardness, the calculated means were found to be the highest in distilled water followed by Renew and Corega while NaOCl showed the lowest hardness values. For pairwise comparisons for all tested properties, most of the pairs were significantly different from each other; however, some pairs had insignificant *P* values which were denoted with the same alphabet (Table 2).

The effects of time on color, hardness, and roughness for each solution are presented in Table 3. With regard to the effect of time, color variation was statistically significant for all solutions (*P* < 0.001). In addition, the difference in color was higher as the immersion duration increased for all solutions and nano-ZrO_2_ concentrations. The effect of immersion time on surface roughness showed an upward trend. Roughness values measured at each immersion time increased significantly compared to its preceding value (*P* < 0.001). However, the surface roughness of specimens immersed in distilled water at 0% and 5% nano-ZrO_2_ concentration did not significantly change (*P*=0.56 & 0.17), respectively. In the case of surface hardness, the baseline value was the highest, and it decreased significantly (*P* < 0.001) as immersion time increased except for distilled water at all concentrations.

The effect of nanofiller concentration on the results after immersion in different solutions at a given time was also analyzed (Table 4) and found to be significant for all groups and tested properties except color change at 180 days for NaOCl (*P*=0.286). The increase in Δ*E* was slight (for each solution) as the nano-ZrO_2_ concentration increased. Furthermore, post hoc comparisons for surface roughness showed only two insignificant pairs among all solutions being tested (NaOCl at 2.5% versus 5% nano-ZrO_2_ at 180 and 365 days). Similarly, post hoc results for surface hardness were not significant for NaOCI and Renew at 2.5% versus 5% nano-ZrO_2_ at *T*_1_ and *T*_2_.

**Figure 3:**
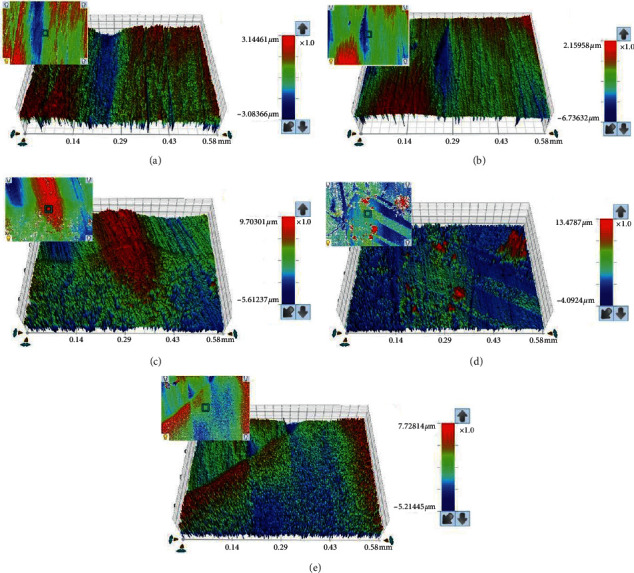
(a-e) Representative surfaces roughness images of unmodified specimens after immersion. (a) Baseline. (b) Distilled water. (c) Corega. (d) NaOCl. (e) Renew.

**Figure 4:**
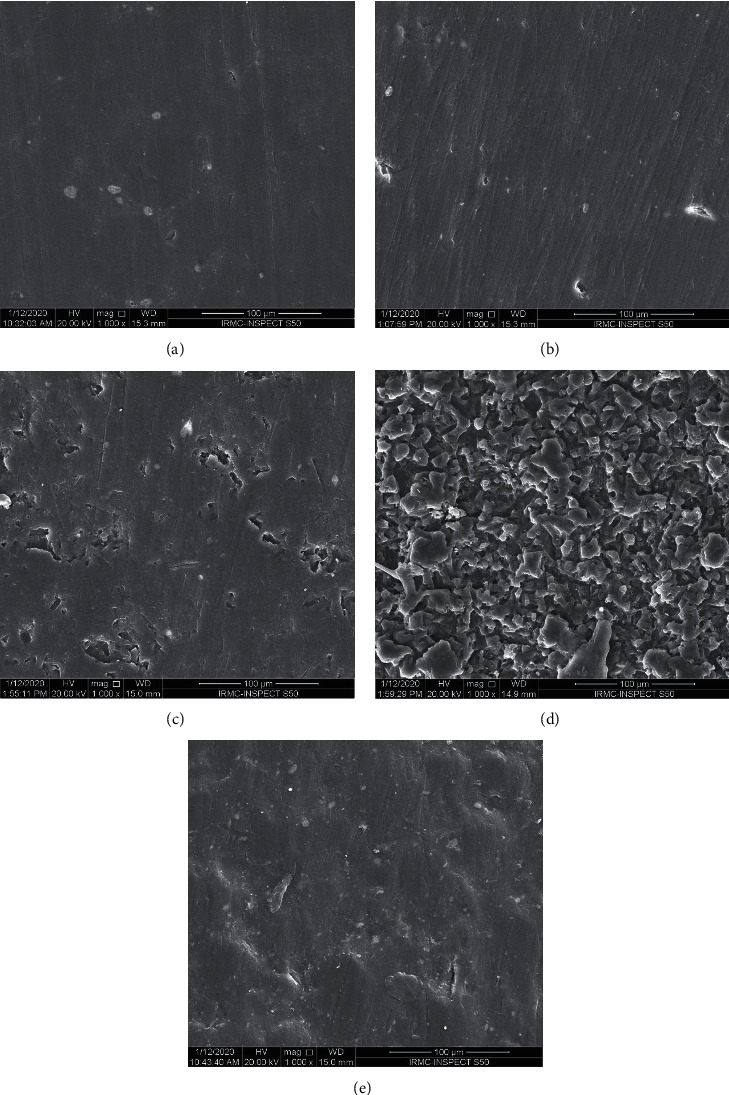
(a–e) Representative SEM for specimen surfaces unmodified specimens after immersion. (a) Baseline. (b) Distilled water. (c) Corega. (d) NaOCl. (e) Renew.

The surface profiles of selected specimens at baseline (*T*_0_), and *T*_2_ for control, Corega, NaOCl, and Renew are shown in Figures 3(a)–3(e), respectively. The surface changes were further confirmed by SEM analysis for all groups (Figures 4(a)–4(e)). The surface in each group showed changes in the topography compared to baseline. At baseline, specimens had smooth surfaces that changed after prolonged immersion. The changes included a higher degree of surface roughness, pores, and voids. The degree of surface changes varied in increasing pattern with immersion in water followed by Renew, and Corega, and the maximum change was seen with NaOCl.

## 4. Discussion

The results of this study revealed significant changes in color, surface roughness, and hardness of PMMA/ZrO_2_ nanocomposite resins after immersion in DCs. Therefore, the null hypothesis was rejected. Based on the results, the immersion of PMMA/ZrO_2_ nanocomposite denture base material for 1 year in distilled water or cleansing solutions caused some color alteration. DCs cause the soluble component and plasticizers to leach out of the denture base resin. Additionally, absorption of water and other salivary components into the resin matrix leads to color change [18]. In the present study, NBS values at *T*_1_ were ≤3 and classified as noticeable but were clinically acceptable. At *T*_2_, NBS values increased and were not consistent for different solutions (water <3, noticeable; Corega and Renew <6, appreciable; and NaOCl >6, much).

Ferracane [19] stated that acrylic resin has a tendency to absorb solvents or water owing to the polarity of the PMMA molecules. The absorbed liquid diffuses into the polymer network and causes hydrolysis and formation of acrylic zones with different optical properties resulting in color change. This might be the reason for color change even with distilled water immersion. Others suggested that the change in color is a result of the whitening action of DCs [16]. Moon et al. [20] and Goiato et al. [21] reported similar results to those of the present study for a long duration of immersion in water. The use of hot water to prepare denture cleansing solutions as recommended by manufacturers may potentiate leaching out of colorants from the resin [16].

Although NaOCl is used for disinfection and biofilm control, drawbacks due to the possibility of whitening were reported and considered as the main disadvantage [22]. This fact is supported by the results of the present study where immersion in NaOCl resulted in significant color changes that were more prominent as the immersion duration increased [9, 23]. Robinson et al. [24] reported that the solvent in denture cleansers penetrates into the polymer network and causes expansion of intermolecular spaces facilitating leaching out of intrinsic pigments and penetration of extrinsic colorants [24]. Thus, this could be the probable reason for color change associated with all test groups at 365 days compared to 180 days or baseline readings. In a study by Lohitha et al. [23], Δ*E* values of acrylic specimens immersed in NaOCl for 90 days were minimal whereas Δ*E* values for 180 days for all groups except control were >12. Contrary to the present study, Paranhos et al. [10] reported no color alteration after immersion in DCs for 20 min and 180 days. Their findings may be due to the use of different resins or a short simulation period.

Corega contains oxygen releasing agents and enzymes, supporting the theory that oxidation combined with an alkaline solution can be detrimental [22]. A previous study [4] showed a significant change in acrylic resin color after immersion in Corega; however, the results turned to be below the threshold of “3” according to the NBS unit which is considered acceptable. Peracini et al. [4] and Hong et al. [9] reported color changes between 0.5 and 2.3 as quantified by NBS after 180 days of immersion. Those conform with the values of the present study. However, in the current study, higher Δ*E* levels were detected after 365 days of immersion. The differences between these two studies [4, 9] and the present study could be due to the difference in immersion duration or composition of resin material. Renew showed the same behavior as Corega but with less detrimental effects, in agreement with a previous study by Al-Thobity et al. [2]. This finding may be attributed to lower peroxide content in Renew compared to Corega [2].

A study reported that immersion in DCs increased the surface roughness of acrylic denture base resin [4]. According to the results of the present study, all solutions increased *R*_a_ and the maximum value was recorded with NaOCl which was confirmed with SEM images indicating its great effect on the specimens. Additionally, the effect of immersion on surface roughness was time-dependent which is in agreement with the results of a previous study [7].

Corega and Renew showed significant *R*_a_ changes with time intervals with no significant difference between them, in agreement with Althobity et al. [2] and contrary to Peracini et al. [4]. The increase in *R*_a_ value in this study could be attributed to soaking temperature, peroxide content, amount of released oxygen, and its possible action in mechanical cleansing of the denture base material [2, 25]. Renew increased surface roughness but to a lesser degree than Corega, which may be associated with its lower peroxide level and lack of mechanical cleaning.

As reported in the literature, *R*_a_ of denture base material was variable after immersion in denture cleansers. Some studies reported that alkaline peroxide-type disinfecting agent [4, 25] and NaOCl [7, 18, 26] increased *R*_a_ of denture base resin while others [6, 25, 27] did not report changes in roughness, which is in disagreement with the results of the present study. These studies had different testing parameters, immersion periods, cleanser concentration, and simulation duration. The increase in *R*_a_ may be linked to surface degeneration due to the effervescent action of perborate content in denture cleanser [28].

With nano-ZrO_2_ addition, *R*_a_ increased and this increase was directly related to the concentration, in agreement with a previous study [29]. All *R*_a_ values were above the clinically acceptable threshold for surface roughness (*R*_a_ = 0.2 *μ*m) except 2.5% immersed in distilled water at *T*_1_ and *T*_2_. This suggests that immersion in denture cleansing solution might alter the material's surface and increase its vulnerability to microbial adhesion [2].

Similar to previous reports [3, 17], surface hardness decreased after immersion in DCs, and this decrease was time-dependent. Diluted NaOCl is absorbed by acrylic resin and may act as a plasticizer with possible alteration in chemical structure or surface integrity [18]. Some authors related that to chemical interactions between acrylic resin and DCs (i.e., chlorine) [26].

Nano-ZrO_2_ improved the hardness of acrylic resin. Furthermore, after immersion, reinforced specimens had a minimal drop in hardness compared to unmodified specimens with an inverse relationship between the reduction in hardness and nanofiller concentration. This positive effect is attributed to the properties of nanoparticles present at the specimen surface. Regardless of the positive effect of nano-ZrO_2_, NaOCl immersion resulted in the lowest hardness values of all modified groups at all-time intervals.

In the present study, all DCs exhibited a change in color, surface roughness, and hardness of PMMA/ZrO_2_ nanocomposite. Generally, the changes were color alteration, increase in surface roughness above clinically acceptable limit, and decrease in surface hardness. Due to these detrimental effects, alteration in concentration or duration of immersion is required for ZrO_2_-modified specimens. The choice of denture cleanser should be based on the chemistry of resin and cleanser. In addition to that, solution preparation and water temperature must follow the manufacturer's recommendations [18].

Corega and Renew showed similar effects in terms of color change, surface roughness, and hardness, while NaOCl had more dramatic changes. Moreover, the pattern of change was the same for modified and unmodified specimens except that changes were less with modified specimens. This finding indicates a similar mechanism of action of DCs with different magnitudes. Nano-ZrO_2_ did not completely prevent the negative effects of DCs compared to unreinforced specimens. In the end, the authors would like to report some of the study limitations. First is the number of cleansing agents, the use of only one type of denture base material, and one type of nanoparticles. Second, the surfaces of the specimens used in this study were flat and did not mimic the surface topography of actual dentures. In addition, the effects of saliva and other aging processes such as dynamic loading and thermal changes were not taken into consideration. Third, although Δ*E*_*ab*_^*∗*^ formula of the CIELab color space is commonly used to quantify the color change of acrylic resin, the new formula (Δ*E*_00_^*∗*^) of the CIEDE2000 with its improvements may be alternatively used for color change quantification. Therefore, further investigations on the color stability of nanocomposite denture base resins using the CIEDE2000 formula are recommended.

## 5. Conclusions

Within the limits of the current study, it may be concluded that Corega, Renew, and sodium hypochlorite denture cleansers negatively affected the color, reduced surface hardness, and increased roughness of unmodified and nano-ZrO_2_-modified denture base materials. Sodium hypochlorite had the most detrimental effects on the studied properties. Color change and roughness had a direct relation with immersion time while hardness had an inverse relation. Therefore, appropriate denture cleansers should be selected based on their chemical structure, concentration, and immersion duration even with enhanced denture bases.

## Figures and Tables

**Table 1:** Cleansing agents, composition, and preparation as recommended by the manufacturer and immersion protocol.

Denture cleansers/manufacturer	Generic description	Composition	Preparation and immersion instructions	Immersion protocol
Distilled water (W)	Distilled water		(i) Immersion for the whole duration of the experiment at room temperature	(i) Baseline measurement was done using specimens with different levels of nano-ZrO_2_ reinforcement immersed in distilled water for 2 days (*T*_0_) (ii) Then, all specimens were immersed in respective solutions for 180 days followed by measurements (*T*_1_). (iii) The immersion continued for further 185 days to simulate 1 year of clinical use and final measurements were made (*T*_2_). (iv) After immersion in solutions, resin specimens were retrieved, thoroughly washed under running water, and stored in distilled water at room temperature until the next immersion cycle the following day. (v) The immersion procedure was performed by one investigator where fresh solutions were prepared daily for 365 days.
Corega (C)/GlaxoSmithKline; Stafford Miller, Ireland; Lot no. XL9X	Disinfectant effervescent tablet	Potassium monopersulfate; sodium bicarbonate; sodium lauryl sulfoacetate; sodium perborate monohydrate; sodium polyphosphate	(i) One tablet dissolved in 200 mL of warm tap water (40°C) (ii) Immersion for 3 minutes	
Sodium hypochlorite-NaOCl (S)	Sodium hypochlorite solution	Sodium hypochlorite solution, 1% active chlorine	(i) Solution of 5.25% sodium hypochlorite (NaOCL, 1:5 dilution) was diluted to obtain 1% sodium hypochlorite by adding 50 mL NaOCl to 200 mL water. (ii) Immersion for 10 minutes at room temperature	
Renew (R)/Mid Continental dental Supply Co. LTD., Winnipeg, Canada; Lot no. 144	Disinfectant effervescent powder	Sodium hypochlorite, sodium bicarbonate; salt and lemon; sodium perborate; sodium phosphate; monosodium persulfate; ethylene diamine tetraacetic acid (EDTA)	(i) One teaspoon dissolved in 200 mL of warm tap water (40°C) (ii) Immersion for 20 minutes	

**Table 2:** Mean and SD values of color (Δ*E*), surface roughness (*μ*m), and Hardness (VHN) of acrylic specimens immersed in different solutions at a given time and concentration.

Nano-ZrO_2_	Time	Water (W)	Corega (C)	NaOCl (S)	Renew (R)	*P* value
		Mean (SE)	Mean (SE)	Mean (SE)	Mean (SE)	
Color						
0%	*T*_1_	1.51 (0.02)	2.98 (0.03)^a^	3.01 (0.05)^a^	1.93 (0.04)	**0.001** ^*∗*^
0%	*T*_2_	1.99 (0.05)	4.19 (0.06)	5.92 (0.09)	3.83 (0.13)	**0.001** ^*∗*^
2.5%	*T*_1_	1.69 (0.02)	2.98 (0.04)	3.05 (0.07)	1.97 (0.02)	**0.001** ^*∗*^
2.5%	*T*_2_	2.04 (0.05)	4.96 (0.05)	6.85 (0.12)	2.99 (0.07)	**0.001** ^*∗*^
5%	*T*_1_	1.45 (0.01)	2.89 (0.08)	3.01 (0.05)	1.96 (0.05)	**0.001** ^*∗*^
5%	*T*_2_	2.19 (0.03)	4.99 (0.08)	6.19 (0.09)	3.19 (0.06)	**0.001** ^*∗*^

Roughness						
0%	*T*_1_	0.14 (0.01)	0.19 (0.02)^a^	0.24 (0.02)	0.18 (0.01)^a^	**0.001** ^*∗*^
0%	*T*_2_	0.14 (0.01)	0.23 (0.01)	0.36 (0.01)	0.20 (0.02)	**0.001** ^*∗*^
2.5%	*T*_1_	0.17 (0.02)	0.23 (0.02)	0.33 (0.02)	0.21 (0.01)	**0.001** ^*∗*^
2.5%	*T*_2_	0.19 (0.02)	0.27 (0.02)^a^	0.39 (0.02)	0.26 (0.02)^a^	**0.001** ^*∗*^
5%	*T*_1_	0.24 (0.02)	0.27 (0.0)^a^	0.34 (0.02)	0.26 (0.01)^a^	**0.001** ^*∗*^
5%	*T*_2_	0.25 (0.01)	0.33 (0.02)	0.39 (0.01)	0.29 (0.02)	**0.001** ^*∗*^

Hardness						
0%	*T*_1_	31.05 (1.3)	27.79 (1.4)^a^	25.17 (1.1)	28.84 (1.1)^a^	**0.001** ^*∗*^
0%	*T*_2_	30.3 (1.3)	25.68 (1.3)^a,b^	24.16 (1.4)^a^	27.29 (1.4)^b^	**0.001** ^*∗*^
2.5%	*T*_1_	38.6 (1.8)^a^	34.85 (1.2)^b^	34.29 (1.3)^b^	39.59 (1.1)^a^	**0.001** ^*∗*^
2.5%	*T*_2_	39.07 (0.97)	33.11 (0.84)	31.54 (1.6)	36.76 (1.1)	**0.001** ^*∗*^
5%	*T*_1_	41.46 (1.5)^a^	40.82 (1.4)^a^	35.22 (1.2)	38.80 (1.2)	**0.001** ^*∗*^
5%	*T*_2_	41.13 (1.3)	37.45 (1.5)^a^	30.28 (1.3)	36.07 (1.2)^a^	**0.001** ^*∗*^

^*∗*^indicates a significant difference at *α* = 0.05. ANOVA and post hoc tests were used for statistical analysis to find statistical significance within each concentration using different solutions; hence, a comparison was done horizontally. Groups with similar letters indicate no significant difference (*P* > 0.05).

**Table 3:** Effect of variation in immersion time on acrylic specimen's color (Δ*E*), surface roughness (*μ*m), and hardness (VHN) after immersion in different cleansing solutions.

Nano-ZrO_2_ (%)	Time	Water (W) Mean (SE)	*P*	Corega (C) Mean (SE)	*P*	NaOCl (S) Mean (SE)	*P*	Renew (R) Mean (SE)	*P*
Color									
0	*T*_1_	1.51 (0.02)	0.001 ^*∗*^	2.99 (0.03)	0.001 ^*∗*^	3.01 (0.05)	0.001 ^*∗*^	1.92 (0.04)	0.001 ^*∗*^
	*T*_2_	1.99 (0.04)		4.91 (0.06)		5.9 (0.09)		3.83 (0.12)	
2.5	*T*_1_	1.69 (0.02)	0.001 ^*∗*^	2.98 (0.04)	0.001 ^*∗*^	3.05 (0.07)	0.001 ^*∗*^	1.97 (0.02)	0.001 ^*∗*^
	*T*_2_	2.04 (0.05)		4.96 (0.05)		6.85 (0.13)		2.99 (0.07)	
5	*T*_1_	1.45 (0.01)	0.001 ^*∗*^	2.89 (0.08)	0.001 ^*∗*^	3.01 (0.05)	0.001 ^*∗*^	1.96 (0.05)	0.001 ^*∗*^
	*T*_2_	2.2 (0.03)		4.99 (0.08)		6.91 (0.09)		3.20 (0.06)	

Roughness									
0	*T*_0_	0.14 (0.02)	0.562	0.14 (0.02)	0.001 ^*∗*^	0.14 (0.02)	0.001 ^*∗*^	0.14 (0.02)	0.001 ^*∗*^
	*T*_1_	0.14 (0.01)		0.19 (0.02)		0.24 (0.02)		0.18 (0.02)	
	*T*_2_	0.14 (0.01)		0.23 (0.01)		0.36 (0.01)		0.20 (0.02)	
2.5	*T*_0_	0.19 (0.01)^a^	0.012 ^*∗*^	0.19 (0.01)	0.001 ^*∗*^	0.19 (0.01)	0.001 ^*∗*^	0.19 (0.01)	0.001 ^*∗*^
	*T*_1_	0.17 (0.02)		0.23 (0.02)		0.33 (0.02)		0.21 (0.01)	
	*T*_2_	0.19 (0.01)^a^		0.27 (0.02)		0.39 (0.02)		0.26 (0.02)	
5	*T*_0_	0.26 (0.02)	0.171	0.26 (0.02)^a^	0.001 ^*∗*^	0.26 (0.02)	0.001 ^*∗*^	0.26 (0.02)a	0.001 ^*∗*^
	*T*_1_	0.24 (0.02)		0.27 (0.0)^a^		0.34 (0.02)		0.26 (0.01)a	
	*T*_2_	0.25 (0.01)		0.33 (0.02)		0.39 (0.01)		0.29 (0.02)	

Hardness									
0	*T*_0_	30.63 (1.68)	0.518	30.63 (1.68)	0.001 ^*∗*^	30.63 (1.68)	0.001 ^*∗*^	30.63 (1.68)	0.001 ^*∗*^
	*T*_1_	31.05 (1.34)		27.79 (1.37)		25.17 (1.06)^a^		28.84 (1.1)^a^	
	*T*_2_	30.3 (1.27)		25.68 (1.3)		24.16 (1.36)^a^		27.29 (1.43)^a^	
2.5	*T*_0_	38.77 (1.42)	0.786	38.77 (1.42)	0.001 ^*∗*^	38.77 (1.42)	0.001 ^*∗*^	38.77 (1.42)^a^	0.001 ^*∗*^
	*T*_1_	38.64 (1.76)		34.85 (1.23)		34.29 (1.26)		39.59 (1.07)^a^	
	*T*_2_	39.07 (0.97)		33.11 (0.84)		31.54 (1.58)		36.76 (1.1)	
5	*T*_0_	41.62 (1.44)	0.722	41.62 (1.44)^a^	0.001 ^*∗*^	41.62 (1.44)	0.001 ^*∗*^	41.62 (1.44)	0.001 ^*∗*^
	*T*_1_	41.46 (1.46)		40.82 (1.4)^a^		35.22 (1.2)		38.8 (1.21)	
	*T*_2_	41.13 (1.27)		37.45 (1.45)		30.27 (1.32)		36.07 (1.2)	

^*∗*^Significant difference at *α* = 0.05. The same letter indicates nonsignificant vertically per concentration (*P* > 0.05). *T*_0_ = 2 days of immersion, *T*_1_ = 180 days of immersion, *T*_2_ = 365 days of immersion, 0% = pure acrylic resin, 2.5%, acrylic resin reinforced with 2.5% nano-ZrO_2_, 5% = acrylic resin reinforced with 5% nano-ZrO_2_.

**Table 4:** Effect of variation in ZrO_2_ concentration on acrylic specimen color (Δ*E*), surface roughness (m*μ*), and hardness (VHN) after immersion in different cleansing solutions for the specific times.

Time	Nano-ZrO_2_ (%)	Water (W) mean (SE)	*P*	Corega (C) mean (SE)	*P*	NaOCl (S) mean (SE)	*P*	Renew (R) mean (SE)	*P*
Color									
*T*_1_	0	1.51 (0.02)	**0.001** ^*∗*^	2.99 (0.03)^a^	**0.001** ^*∗*^	3.01 (0.05)^a^	**0.286**	1.92 (0.04)	**0.009** ^*∗*^
	2.5	1.69 (0.02)		2.98 (0.04)^a^		3.05 (0.07)^a^		1.97 (0.02)^a^	
	5	1.45 (0.01)		2.89 (0.08)		3.01 (0.05)^a^		1.96 (0.05)^a^	
*T*_2_	0	1.99 (0.04)^a^	**0.001** ^*∗*^	4.91 (0.06)^a^	**0.04** ^*∗*^	5.9 (0.09)	**0.001** ^*∗*^	3.83 (0.12)	**0.001** ^*∗*^
	2.5	2.04 (0.05)^a^		4.96 (0.05)^a,b^		6.85 (0.13)^a^		2.99 (0.07)	
	5	2.20 (0.03)		4.99 (0.08)^b^		6.91 (0.09)^a^		3.20 (0.06)	

Roughness									
*T*_0_	0	0.14 (0.02)	**0.001** ^*∗*^						
	2.5	0.19 (0.01)							
	5	0.26 (0.02)							
*T*_1_	0	0.14 (0.01)	**0.001** ^*∗*^	0.19 (0.02)	**0.001** ^*∗*^	0.24 (0.02)	**0.001** ^*∗*^	0.18 (0.02)	**0.001** ^*∗*^
	2.5	0.17 (0.02)		0.23 (0.02)		0.33 (0.02)^a^		0.21 (0.01)	
	5	0.24 (0.02)		0.27 (0.0)		0.34 (0.02)^a^		0.26 (0.01)	
*T*_2_	0	0.14 (0.01)	**0.001** ^*∗*^	0.23 (0.01)	**0.001** ^*∗*^	0.36 (0.01)	**0.001** ^*∗*^	0.20 (0.02)	**0.001** ^*∗*^
	2.5	0.19 (0.01)		0.27 (0.02)		0.39 (0.02)^a^		0.26 (0.02)	
	5	0.25 (0.01)		0.33 (0.02)		0.39 (0.01)^a^		0.29 (0.02)	

Hardness									
*T*_0_	0	30.63 (1.68)	**0.001** ^*∗*^						
	2.5	38.77 (1.42)							
	5	41.62 (1.44)							
*T*_1_	0	31.05 (1.34)	**0.001** ^*∗*^	27.79 (1.37)	**0.001** ^*∗*^	25.17 (1.06)	**0.001** ^*∗*^	28.84 (1.1)	**0.001** ^*∗*^
	2.5	38.64 (1.76)		34.85 (1.23)		34.29 (1.26)^a^		39.59 (1.07)^a^	
	5	41.46 (1.46)		40.82 (1.4)		35.22 (1.2)^a^		38.8 (1.21)^a^	
*T*_2_	0	30.30 (1.27)	**0.001** ^*∗*^	25.68 (1.3)	**0.001** ^*∗*^	24.16 (1.36)	**0.001** ^*∗*^	27.29 (1.43)	**0.001** ^*∗*^
	2.5	39.07 (0.97)		33.11 (0.84)		31.54 (1.58)^a^		36.76 (1.1)^a^	
	5	41.13 (1.27)		37.45 (1.45)		30.27 (1.32)^a^		36.07 (1.2)^a^	

^*∗*^Significant difference at *α* = 0.05. The same letter indicates nonsignificant vertically per concentration (*P* > 0.05). *T*_0_ = 2 days of immersion, *T*_1_ = 180 days of immersion, *T*_2_ = 365 days of immersion, 0% = pure acrylic resin, 2.5%, acrylic resin reinforced with 2.5% nano-ZrO_2_, 5% = acrylic resin reinforced with 5% nano-ZrO_2._ The same small letters indicated nonsignificant vertically per time.

## Data Availability

The data used to support the findings of this study are included within the article, and necessary explanations in relation to this can be obtained from the corresponding author upon request.
